# Association between long COVID, functional activity, and health-related quality of life in older adults

**DOI:** 10.1186/s12877-023-03757-w

**Published:** 2023-01-23

**Authors:** Sanaz Shanbehzadeh, Nasibeh Zanjari, Marzieh Yassin, Zeynab Yassin, Mahnaz Tavahomi

**Affiliations:** 1 Rehabilitation Research Center, Department of Physiotherapy, School of Rehabilitation Sciences, Iran University of Medical Sciences (IUMS), Tehran, Iran; 2 Iranian Research Center on Aging, University of Social Welfare and Rehabilitation sciences, Tehran, Iran; 3 Hazrat Rasool Hospital, Iran University of Medical Sciences (IUMS), Niayesh St., Sattarkhan Ave., Tehran, Iran

**Keywords:** Long COVID, Quality of life, Aging, Coronavirus, Rehabilitation

## Abstract

**Background:**

Older adults experience persistent symptoms post-COVID-19, termed as Long COVID, affecting their physical and mental health. This study aimed to evaluate the effects of Long COVID, level of physical activity, and functional decline on older adults’ health-related quality of life post-COVID-19.

**Methods:**

This cross-sectional study was conducted on 121 older adults with 60 to 90 years old post-coronavirus infection. The standardized metrics used in the study were Fatigue Severity Scale, Physical Activity Elderly, SF12, Post-COVID-19 functional status scale, and COVID-19 Yorkshire rehabilitation screening scale. The severity of coronavirus infection was evaluated by changes in chest CT scan images and O_2_ saturation at hospital admission. Data were analyzed using linear regression analyses.

**Results:**

The results of regression analysis revealed six factors to be predictors of physical health at 6 months post-COVID-19 (F = 9.046, *P* < 0.001; explained variance 63%), which the significant factors were fatigue, level of physical activity, worsened pain, difficulties in activities of daily living and cognitive-communication problems. Among these factors, greater fatigue and worsened pain intensity were the strongest predictors. Mental health was associated with days of hospitalization and cognitive-communication problems (F = 2.866, *P* < 0.001; explained variance 35%).

**Conclusions:**

Considering the negative impact of fatigue, pain, low physical activity, and cognitive-communication problems on health-related quality of life, early and accurate evaluation and management are required for recovered older adults post-COVID-19.

## Background

The novel coronavirus SARS-CoV-2 (COVID-19) disease has been a global pandemic since March 2020. COVID-19 symptoms commonly manifest as fever, myalgia or arthralgia, headache, fatigue, and shortness of breath that could lead to severe multiple organ failure [1]. All age groups can become infected with the new coronavirus strain. However, older adults are more susceptible to severe illness due to underlying comorbidities such as diabetes, hypertension, and cardiovascular diseases [2].

Long COVID is defined as persistent symptoms experienced in recovered patients from COVID-19 causing of substantial disease burden [3]. Persistent symptoms such as fatigue, pain, shortness of breath, and mental health problems have been reported in COVID-19 survivors [3, 4]. Several studies have reported an association between Long COVID and health-related quality of life (HRQoL) at 6-to-12-month post-COVID-19 [5, 6]. HRQoL as a subdivision of quality of life that encompasses the physical, emotional, and social components associated with an illness [7]. Physical aspect of HRQoL is related to the functional status, independence and how well they are engaged in daily activities [8]. The mental aspect includes, the internal subjective perceptions, such as vitality, pain, and symptoms of depression and anxiety, and general health [7]. In addition to persistent symptoms, social distancing and hospitalization could affect older adults’ daily activity and physical functioning which could adversely impact HRQoL [9]. Since most hospitalized patients with coronavirus infection were adults over 60 years [10], functional activity and independence in daily activities are important determinants of HRQoL for this population [8].

HRQoL has been considered an essential factor for examining the impact of diseases on the physical, mental, and social domains of patients’ health [11]. In order to plan an efficient rehabilitation program, measuring HRQoL and the factors associated with it is of great importance to improve their lives in the future. HRQoL in older adults is a significant public health issue; thereby, research into older adults’ physical and mental health is vital for maintaining active aging [12]. Poor HRQoL is associated with higher morbidity and mortality rates and greater use of health care services [13]. Therefore, HRQoL can be used as a metric to assess the impact of a chronic condition on their daily lives. In order to plan an efficient rehabilitation program, it is essential to find out which persistent symptom is primarily related to HRQoL.

Although studies have evaluated HRQoL in COVID-19 survivors, no study has considered the association between Long COVID, physical activity, and daily function on HRQoL in older adults. Since reduced HRQoL has been reported in older adults at six-month post coronavirus hospitalization [14], assessment of factors associated with HRQoL is of great importance.

To our knowledge, the long-term consequences of COVID-19 affecting HRQoL in the older adult population are largely unknown. Therefore, this study aimed to assess the association between Long COVID, level of physical activity, and functional decline on older adults’ HRQoL post-COVID-19.

## Methods

In this cross-sectional single-center study, older adults who were sufficiently ill to require hospitalization between March 1st to August 30th, 2020, at Hazrat Rassol Hospital located in Tehran the capital city of Iran, were contacted on average 6 months post-hospital discharge (September 2020 to February 30th, 2021. From 309 discharged older adults, 27 patients had died within the 6 months following admission, and 161 were demented, bedridden, unreachable, declined to participate, or were non-Farsi-speaking patients. Finally, 121 older adults completed the questionnaires. Their diagnosis was based on confirmed acute respiratory syndrome coronavirus (SARS-CoV-2) infection by a positive result on polymerase chain reaction testing of nasopharyngeal samples. To define “older adults,” we used the cut-off age of 60 years, as suggested by the World Health Organization (WHO) [15].

Disease severity was defined in four grades according to the WHO criteria for patients’ chest computed topography (CT) scan images on the initial scan obtained at admission [16]. This was based on the severity of changes in each lung zone (S0 = without lung involvement, S1 = involvement of maximum two lobes of lung with ground-glass opacity, nodule or consolidation with less than one-third of each lobe, S2 = involvement of three or four lobes of lung with less than one-third or involvement of one or two lobes with more than one third, S3 involvement of five lobes of the lung with less than one-third or involvement of three lobes with more than one third, S4 = more lung involvement. In addition, the percent of O_2_ saturation (moderate disease: SpO_2 ≥_ 90%, severe disease: SpO_2_ < 90%) at admission was accounted in the analysis [17].

### Development of the telephone screening tool

A COVID-19 rehabilitation a series of standardized and validated questionnaires: (I) Sociodemographic data were collected (sex, age, education, working status, and household members), (II) Fatigue Severity Scale (FSS), (III) Physical Activity Scale for Elderly (PASE), (IV) Quality of life with Short-Form Health Survey (SF12), (VI) Functional status was assessed with Post-COVID-19 Functional Status (PCFS) Scale and (VII) Screening COVID-19 Yorkshire Rehabilitation Scale (C19-YRS) [18].

In order to minimize face-to-face interaction with older adults who are susceptible to coronavirus infection, the package of questionnaire survey was constructed and administered using an online survey. Iranian platform (Porsline; https://survey.porsline.ir) was used, and we provided participants with web links, and the responses to the questionnaires were automatically stored in a platform. All participants were asked to complete the survey using their mobile phones at 6 months after hospital discharge. For those older adults who had difficulties in mobile phone, their family members were invited to assist them in completing the online survey. In order to accurately complete the questionnaires by the elderly, a trained interviewer contacted all eligible patients by phone and completed interviewer-assisted over telephone.

Post-COVID-19 symptoms were asked by C19-YRS, that includes two parts of symptom severity and functional disability [18, 19]. Two separate visual analog scales (VAS) for pre- and post-COVID-19 were used for each item. a) Participants were asked to rate any new, remaining, or worsened post-COVID-19 symptoms from before coronavirus infection (shortness of breath during daily activity, fatigue, and pain), b) Functional disability that asks about perceived post-COVID-19 problems with mobility/walking, usual care, daily activity and economic status as compared to pre-infection. The changes observed in the variables between pre- and post-COVID-19 ratings on VAS (VAS scores for post-infection minus pre-infection score) were considered the impact of COVID-19. C19-YRS also includes an item related to communication problems. This item is mainly related to cognitive-communication problems rated on the VAS Likert scale, which asks about any problems that have been felt with auditory and reading comprehension, putting thoughts into words, and difficulty with having conversation post-coronavirus infection.

The Persian version of SF-12 that has acceptable reliability and validity was used to measure HRQoL [20] and distinguishes a variety of health statuses among older adults [21]. SF-12 contains eight dimensions that form two subscales, the Physical Component Scale (PCS) and Mental Component Scale (MCS). Scores were calculated according to the SF-12 scoring algorithm proposed by John E. Ware in 1995. All summary scores range from 0 to 100, comparable with SF-36 scores, where higher scores indicate better HRQoL [22].

The PASE has acceptable psychometric properties in Persian-speaking older adults [23]. It includes ten items for evaluating the level of physical activity over 1 week. PASE scores are calculated from weights and frequency values for 12 types ranging from 0 to 400. Physical activity levels were categorized based on PASE score into 4 levels. Level 1: including PASE ≤93, level 2: PASE = 94–146, level 3: PASE = 147–206 and level 4: PASE ≥206 [24].

Fatigue was assessed using the Persian version of FSS which has acceptable psychometric properties [25], which consists of nine statements that attempt to evaluate the impact of the severity of fatigue and its effect on the person’s activities over the past week. The participants were asked to read each statement and circle a number from 1 (strong disagreement) to 7 (strong agreement), which scores ranging from 7 to 63. The scoring is the mean value of the nine items, and a score of ≥4 is considered severe fatigue [26].

The Persian valid PCFS was used to assess the level of functional status impairment post-COVID-19 [27], which is composed of five scale grades: grade 0 (No functional limitations); grade 1 (Negligible functional limitations); grade 2 (Slight functional limitations); grade 3 (Moderate functional limitations) and grade 4 (Severe functional limitations) [28].

### Ethical approval

The study was approved by the Ethics Committee at Iran University of Medical Sciences (IR.IUMS.REC.1399.1044) and deemed to comply with the Declaration of Helsinki. After explaining the purpose of the research, verbal informed consent was obtained for all participants (if the subjects are illiterate informed consent was obtained from legal representative.)

### Statistical analysis

Data analysis was undertaken using SPSS 20.0 software. Descriptive statistics, such as frequency, percentage, mean, and standard deviation (SD), were conducted to illustrate the demographic and clinical characteristics and the self-report impact of COVID-19 in this study sample. Bivariate analysis was done to investigate the significant association between PCS and MCS scores. Pearson’s correlation was performed for continuous variables, and for categorical variables, the independent samples *t*-test and analysis of variance (ANOVA) were used to test the group differences in means of PCS and MCS score. Multiple linear regression analysis was used to determine independent variables associated with PCS and MCS dimensions of SF-12. A *p*-value threshold of < 0.05 was considered statistically significant.

The sample size was calculated with G*Power, version 3.1.9.2 for a linear multiple regression model with an effect size f2 of 0.12, the number of tested predictors as 2, and the total number of predictors as 19, an alpha = 0.05, and the desired power of 90%, the required sample size was 110, with considering 10% for not completing the interview 121 individuals were included. In addition, for prevalence of symptoms the minimum required sample size was estimated to be 121 using the Cochran formula, *N* = (Zα) ^2^ P (1 − P) /d^2^ taking into account the prevalence of 28% for fatigue (*p* = 0.28) [5], confidence interval of 95% and precision (e) of 0.08.

## Results

From 309 discharged older adults, 121 patients completed the questionnaires on average 6 months post-hospital discharge. The mean (SD) age was 70.69 (6.73), the length of stay in the hospital 7.56 (6.02), and the time since discharge was 198 (49.48). Comorbidity was present in 70%; the most common comorbidities were hypertension (40.8%), cardiovascular disease (24.2%), and diabetes (35%). Approximately 16% of the sample were living alone. Moreover, 24% of the sample had no formal education, and 48.8% had primary education (Table 1). Approximately 48% of patients, at hospitalization admission, had SpO_2_ < 90% (severe disease). Based on CT scan findings, 56.2% had grade 3 severity. Considering the impact of COVID-19 on their functional status, 23.3% report slight to severe limitations. Around 72% of COVID-19 survivors report long-lasting clinical complications. The frequently reported post-discharge symptoms were asthenia/fatigue (47.9%), pain (joint or muscle pain) (35.5%), shortness of breath (34.7%), and mental health problems (14.87%).

**Table 1:** The mean (SD) and percent of the study variables

	Mean (SD)	N (%)
Sex	Male	60 (50.4)
	Female	61 (49.6)
Age categories	Youngest old (60–74)	91 (75.2)
	Old (75–84)	25 (20.7)
	Oldest old (85+)	5 (4.1)
Age mean (SD)	70.69 (6.73)	
length of stay	7.56 (6.02)	
Days since hospital admission	198 (49.48)	
Living arrangement	Living Alone	20 (16.5)
	Not alone	101 (83.5)
Educational level	Illiterate	29 (24.0)
	Primary or Elementary	59 (48.8)
	Diploma	19 (15.7)
	higher education	14 (11.7)
The percent of the study Clinical Characteristic		
Number of comorbidities	0	32 (26.4)
	1	53 (43.0)
	2	21 (17.4)
	3+	16 (13.2)
SpO_2_	≥90%	62 (51.2)
	<90%	58 (47.9)
Disease severity (CT scan)	S1	17 (14.0)
	S2	32 (26.4)
	S3	68 (56.2)
	S4	4 (3.3)
Self-reported impact of covid-19		
Effect of Covid-19 on Functional status	No limitation	53 (44.2)
	Negligible	39 (32.5)
	Slight	14 (11.7)
	Moderate	6 (5)
	Severe	8 (6.7)
Report Post-discharge problems		87 (72.5)
Changes in breath in daily activities		42 (34.7)
Changes in walking		37 (30.6)
Changes in self-care		12 (9.9)
Changes in activities of daily living		53 (43.8)
Changes in Feeling pain		43 (35.5)
Communication problem		36 (29.8)
Feeling fatigued		57 (47.1)
Changes in economic status.		37 (30.6)
The mean (SD) health-related quality of life, fatigue severity and level of physical activity		
PCS	44.70 (11.36)	
MCS	38.00 (8.18)	
Fatigue severity	22.71 (12.34)	
Level of Physical Activity	48.75 (44.15)	

Table 1 shows the self-reported long-lasting effect of COVID-19. Approximately 44% of the COVID-19 survivors perceived problems in their activities in daily living, mobility, and worsened or new pain and shortness of breath compared to pre-coronavirus infection.

The PCS achieved a higher mean score rather than the MCS. Severe fatigue was found in 16.5, and 86.77% of older adults had low physical activity.

Table 2 demonstrates the association of each demographic and clinical characteristic with MCS and PCS. The results showed significantly lower PCS scores in older adults with worsened pain levels, shortness of breath, and difficulty with mobility/walking, self-care, and daily activities. Also, older adults with higher fatigue levels, lower physical activity, and greater cognitive-communication problems had lower PCS scores. Moreover, MCS scores were lower in adults with worsened shortness of breath in daily living, difficulties with self-care and daily activities, economic problems and greater communication problems, and higher levels of fatigue.

**Table 2:** Comparison of SF12 dimensions (PCS and MCS) in variable groups

Characteristic	Group	MCS Mean	Statistics	PCS Mean	Statistics
Sex	Male	38.28	t = 0.37	43.04	t = 0.10
	Female	37.71		46.38	
Age categories	Youngest old (60–74)	37.96	F = 0.90	45.28	F = 0.64
	Old (75–84)	37.23		42.40	
	Oldest old (85+)	42.61		45.49	
Living arrangement	Living alone	37.92	t = −0.045	41.34	t = −1.45
	Not alone	38.01		45.36	
Educational level	Illiterate	39.57	F = 0.934	40.96	F = 0.24
	Primary/Elementary	37.65		45.72	
	Diploma	35.82		45.92	
	Higher education	39.16		46.49	
Length of stay in hospital			*r* = −0.13		*r* = −0.09
Days since hospital admission			*r* = 0.09		*r* = −0.02
Condition of comorbidity			*r* = 0.02		*r* = −0.01
SpO_2_	>90%	38.16	0.93	45.74	0.43
	<90%	38.03		44.09	
CT scan grade	S1	40.46	F = 1.05	34.88	F = 1.41
	S2	46.12		38.27	
	S3	45.22		38.35	
	S4	42.51		43.13	
Post-Covid-19 functional status	No limitation	37.39	F = 1.57	45.59	F = 0.80
	Negligible	40.00		42.33	
	Slight	37.60		44.84	
	Moderate	38.10		47.20	
	Severe	32.62		48.48	
Changes in shortness of breath	No	38.97	t = 2.69**	45.95	t = 2.48*
	Yes	34.07		39.64	
Changes in mobility/walking	No	38.32	t = 0.65	48.46	t = 6.31***
	Yes	37.27		36.16	
Changes in self-care	No	38.55	t = 2.27*	46.17	t = 4.65***
	Yes	32.99		31.31	
Changes in daily activities	No	39.72	t = 2.68**	50.69	t = 8.17***
	Yes	35.79		37.01	
Changes in pain	No	38.45	t = 0.81	49.71	t = 8.11***
	Yes	37.18		36.60	
Changes in economic status	No	39.22	t = 2.53*	45.07	t = 0.54
	Yes	35.22		43.84	
Cognitive communication			*r* = −0.44***		*r* = −0.23*
Fatigue			*r* = −0.24**		*r* = −0.56***
Physical activity total			*r* = 0.06		*r* = 0.34***

F = one-way ANOVA, t = independent-samples t-test, r = Pearson correlation

*PCS* Physical component scale, *MCS* Mental component scale

* *P* ≤ 0.05, ** *P* < 0.01, ***P < 0.001

Table 3 provides a summary of the linear multiple regression analysis conducted to estimate the effects of predictors on PCS and MCS scores of HRQoL after controlling demographic (sex, age, living arrangement, educational level) and clinical variables (number of comorbidities, COVID-19 disease severity, and length of stay in hospital and time since hospital discharge).

**Table 3:** Beta coefficients from the linear regression results of PCS and MCS (*N* = 121)

Variables		PCS	MCS
Fatigue		−0.366**	0.064
Physical activity		0.146*	−0.013
Gender (Men^(R)^)		−0.022	0.061
Age (per year)		0.011	0.001
Living arrangement (alone^(R)^)		0.083	−0.075
Educational level		0.172*	−0.127
length of stay in hospital (per day)		0.020	−0.097*
Days since hospital admission (per day)		0.088	0.225
Number of comorbidities		0.055	−0.023
SpO2 (disease severity < 90%)		0.126	−0.099
CT scan (disease severity per grade)		−0.008	0.162
Post-Covid-19 on functional status (functional limitation per grade)		0.048	−0.051
Changes in shortness of breath (No®)		0.030	−0.058
Changes in mobility/walking (No®)		−0.110	0.100
Changes in self-care (No®)		−0.117	0.111
Changes in activities of daily living (No®)		−0.215*	−0.115
Changes in pain (No®)		−0.318***	0.072
Changes in economic status (No®)		0.007	−0.140
Felling problem in communication (No®)		0.328**	−0.576***
Model Goodness of Fit	B constant	42.98^***^	31.626^**^
	F-value	9.046^***^	2.866^***^
	R	0.794	0.592
	R2 (%)	**63.0**	**35.0**

*PCS* Physical component scale, *MCS* Mental component scale

* *P* < .05; ** *P* < .01; ***P < .001

The multiple linear regression analysis results revealed higher fatigue, lower physical activity levels, worsened daily activities and pain, and cognitive communication problems, significantly contributing to lower PCS scores. From demographic characteristics, only levels of education were correlated with PCS scores. A significant overall model emerged (F = 9.046, *P* < 0.001), explaining around 63% of PCS variance. Among these predictors, fatigue was the strongest predictor of PCS.

Moreover, for MCS, a significant overall model (F = 2.866, *P* < 0.001) explained 35% of the variance, which indicated that cognitive-communication problems and days since hospital admission contributed to lower MCS scores. Among these predictors, cognitive-communication problems were the strongest predictor of MCS.

Table 4 represents the association between disease severities categorized based on CT scan findings with changes in symptoms, usual care, mobility, and daily activities and feeling problem with communication and level of fatigue and physical activity. Only changes in shortness of breath during daily activities were negatively associated with disease severity based on CT findings. Changes in shortness of breath were positively associated with changes in mobility, usual care, and activities of daily living. Fatigue severity was related to changes in shortness of breath, pain, changes in function (self-care, mobility, and activities of daily living), and level of physical activity. Fatigue was also positively related to changes in economic status.

**Table 4:** Correlation between independent study variables and disease severity (*N* = 121)

	1	2	3	4	5	6	7	8	9	10	11
1. Disease severity CT scan	1	−.106	−.233^**^	.069	−.209^*^	−.083	−.022	−.113	.069	−.131	.106
2. Effect of Covid-19 on Functional status	–	1	−.061	.045	.102	.005	.046	.062	−.017	.006	.081
3. Changes in breath during daily activities	–	–	1	.165	.251^**^	.271^**^	.324^**^	.383^**^	.120	.420^**^	−.081
4. Changes in walking/mobility	–	–	–	1	.260^**^	.607^**^	.407^**^	.205^*^	.066	.392^**^	−.186^*^
5. Changes in self-care	–	–	–	–	1	.320^**^	.389^**^	.593^**^	.080	.644^**^	−.260^**^
6. Changes in daily activities	–	–	–	–	–	1	.493^**^	.325^**^	.173	.535^**^	−.399^**^
7. Changes in Feeling pain	–	–	–	–	–	–	1	.351^**^	.144	.573^**^	−.089
8. Changes in economic status	–	–	–	–	–	–	–	1	.194^*^	.682^**^	−.146
9. Cognitive-communication problems (score)	–	–	–	–	–	–	–	–	1	.119	−.011
10. Fatigue severity	–	–	–	–	–	–	–	–	–	1	−.258^**^
11. Level of physical activity	–	–	–	–	–	–	–	–	–	–	1

* *P* < .05; ** *P* < .01; ***P < .001

## Discussion

The present study evaluated the association between persistent physical symptoms of COVID-19 (fatigue, pain, and shortness of breath), cognitive-communication problems, functional mobility, activities of daily living, and level of physical activity with physical and mental health dimensions of HRQoL in older adults recovered from coronavirus infection. The results showed that higher fatigue, lower levels of physical activity, worsened pain, difficulty with daily activities, and lower educational levels were associated with poor physical health at 6 months post-hospital discharge. Mental health was only associated with length of stay in the hospital and cognitive-communication problems post-COVID-19. Physical and mental health aspects of HRQoL were not associated with sex, SpO_2_ at admission, the number of comorbidities, functional status, or abnormal chest CT scan findings at admission.

Poor HRQoL was present in older adults post-COVID-19, indicated by low physical and mental health scores compared to Iranian population norms, particularly in the mental health domain [12]. Consistent with a systematic review study that reported lower quality of life in individual’s post-coronavirus infection [14]. Several studies have emphasized coronavirus infection’s profound effect on older individuals’ mental health [29, 30]. Since older adults are aware of their vulnerability to the coronavirus infection, they might fear its prognosis and complications, which could contribute to fear and anxiety related to death [31]. Moreover, hospitalization would lead to reduced self-confidence, decreased activity, mobility, and physical dependency [32], probably making them more prone to stress, anxiety, and depression [31, 33].

Declined mental health was associated with cognitive-communication problems post-COVID-19. The results showed that the prevalence of cognitive-communication problems was 36% among older adults post-COVID-19. In this regard, previous studies have found impaired immediate verbal memory, delayed verbal memory, verbal fluency, concentration problems, and difficulty with finding words post-COVID-19 [34–36]. Alongside, the probability of the direct neurotropic effect of SARS-CoV-2 on cognitive function, social isolation, and mental problems related to COVID-19 might have affected their concentration on communication [37, 38]. It is possible that communication problems might interfere with older adults’ interpersonal relationships, which is an important factor for social interaction and better HRQoL. Several studies have supported the association between family and interpersonal relationships with HRQoL [39, 40].

Fatigue was the most important predictor of physical health and the most common problem reported by older adults post-COVID-19 infection. The relatively high regression coefficient for the fatigue severity suggests that this may have a greater impact on overall HRQoL than other symptoms. A significant impact of fatigue on the HRQoL has been reported in previous studies in younger populations [41, 42] as well and suggested early monitoring of fatigue and its associated symptoms in post-COVID-19 survivors [42]. Fatigue has been recognized as one of the most disabling complaints in individuals recovered from coronavirus infection [43]. Fatigue could negatively impact physical activity and functional mobility, leading to a vicious cycle of losing physical strength, muscle atrophy, and cardiopulmonary capacity in older adults [44]. Moreover, we found moderate association between low physical activity and feeling difficulties with activities of daily living with fatigue severity. Soyure et al. (2011) indicated that older adults who are physically active are shown to be less fatigued [45]. Almost half of older adults in the preset study reported becoming fatigued more quickly than pre-coronavirus infection (47%). Regardless of the coronavirus infection, fatigue is a significant health problem in older adults [46], and the addition of post-COVID-19 fatigue could exacerbate the condition. The prevalence of fatigue coronavirus infection was higher than the 10% prevalence reported in previous studies in healthy elderly adults [44]. Several central and peripheral factors have been advocated for long-lasting fatigue in post-COVID-19 survivors. In this regard altered CNS activity due to systemic inflammation and cell-mediated immune mechanisms, and peripheral factors such as damage and inflammation of muscle fibers due to infection has been proposed [47]. In addition, psychosocial factors have also been linked associated to chronic fatigue as a consequence of the COVID-19 pandemic [47].

Another factor associated with poor physical health domain of HRQoL was low physical activity and feeling difficulties with activities of daily living post-COVID-19. Inactivity, especially in the post-discharge period, may negatively impact the HRQoL in older adults’ post-hospitalization [48]. In line with our findings, positive associations between physical activity and HRQOL have been previously reported in community-dwelling older adults [49]. Almost 85% of older adults in the present study showed low physical activity. This inactivity could be due to the disease’s side effects or as a result of social distancing, which can lead to greater declines in neuromuscular and cardiorespiratory functions and predisposes older adults to risk of chronic diseases [50, 51]. Physical activity has been considered a critical factor in promoting perceived HRQoL [52, 53]. Hill et al. (2011) found that older adults would increase their participation in exercise if clinicians had recommended them at the hospital; thereby, physical activity should be emphasized during hospitalization [54].

Poor physical health was also related to worsened or new pain post-COVID-19 in older adults. The risk of developing chronic pain has been reported to be higher in older adults post-COVID-19 [55]. This finding was consistent with the study of Sahin et al. that reported lower physical health in recovered individuals with persistent pain post-coronavirus infection [56]. Furthermore, Ojeda et al. reported new pain in half of the critical COVID-19 survivors at one-month post-hospital discharge in severe COVID-19 survivors, which pain was associated with a significantly worse HRQoL [6]. However, none of these studies were conducted specifically on older adults. The present study demonstrates that even after 6 months, 35.5% of older adults reported new or worsened post-COVID-19 pain. This finding was consistent with previous studies reporting long-lasting pain in 4 to 36% of individuals post-COVID-19 [5]. So early biopsychosocial approaches toward pain management, including psychological and physical therapy interventions, can potentially reduce the risk of long-term pain and promote HRQoL [55].

Furthermore, the level of physical activity, and new or worsened symptoms, were not associated with disease severity, except for shortness of breath. Accordingly, previous studies have also reported persistent long-standing symptoms in both severe and mild cases [57]. Moreover, Townsend et al. found no association between fatigue and disease severity 3 months post-coronavirus infection [58]. In addition to pulmonary rehabilitation, individuals with post-COVID-19 have shown benefits from comprehensive rehabilitation programs, including aerobic and strengthening exercises [59, 60].

From all demographic and socioeconomic characteristics variables, only educational level was associated with the physical health dimension of HRQoL. Older adults with higher levels of education had perceived better physical health. Education can aid older adults in acquiring knowledge on post-COVID-19 symptoms and complications and the required personal hygiene, food intake, and level of physical activity [61, 62]. Considering the effect of culture on HRQoL, the results of this correlational study should be interpreted cautiously since different cultures may value aspects of their HRQoL differently. Therefore, treatments for reducing pain levels may change overall HRQoL in one culture, whereas changing fatigue may promote overall HRQoL in another culture [63].

## Limitations

Some limitations may have affected this study, including the possibility of recall bias due to the questions related to the change in the patient’s condition compared to before the coronavirus infection. This study may also have a selection bias because older adults with severe cognitive impairment and disability post-COVID-19 and older adults with severe persistent symptoms (long COVID) could not participate in the interview. Finally, the cross-sectional design of this study does not allow for any inferential explanation of the causal pathway between worsened symptoms and functional and physical decline with HRQoL and its determinants.

## Conclusion

The present study’s found association between post-COVID-19 of fatigue severity, and new or worsened pain with poor physical health. These findings highlight the importance of evaluating and treating post-COVID-19 fatigue and pain, which are comprehensive terms that depend on multiple conditional, cognitive, physiological, and psychological factors. Therefore, a standard assessment approach may be required for appropriate rehabilitation for recovered older adults post-COVID-19. Moreover, cognitive-communication problems were related to poor mental health, hence, diagnostic and intervention approaches should begin as early as possible for cognitive-communication deficiencies as an essential component of rehabilitation programs.

## Data Availability

The datasets used and/or analyzed during the current study are available from the corresponding author upon reasonable request.

## Abbreviations

COVID-19: Coronavirus Disease
HRQoL: Health-related Quality of Life
FSS: Fatigue Severity Scale
PASE: Physical Activity Scale for Elderly
SF12: Quality of life with Short-Form Health Survey
PCFS: Post-COVID-19 Functional Status
C19-YRS: COVID-19 Yorkshire Rehabilitation Scale
VAS: Visual Analogue Scale
PCS: Physical Component Scale
MCS: Mental Component Scale
SD: Standard Deviation
ANOVA: Analysis of Variance
WHO: World Health Organization
